# Utility of 3D Imaging in the Objective Evaluation of Glabellar Lines Following Botulinum Toxin Treatment

**DOI:** 10.3390/diagnostics16050679

**Published:** 2026-02-26

**Authors:** Chenhui Yan, Chenyu Huang, Dian Chen, Xiaoming Hu, Jie Ren, Yi Zhao

**Affiliations:** 1 Department of Dermatology, Beijing Tsinghua Changgung Hospital, School of Clinical Medicine, Tsinghua University, Beijing 102218, China; yanchenhui96@163.com (C.Y.);; 2 Photomedicine Laboratory, Institute of Precision Medicine, Tsinghua University, Beijing 100084, China; 3 School of Life Science, Beijing Institute of Technology, Beijing 100081, China

**Keywords:** glabellar lines, objective assessment, quantitative evaluation, three-dimensional imaging, wrinkle depth

## Abstract

**Background/Objectives:** Objective and reproducible evaluation of glabellar lines remains challenging, as current clinical assessments rely largely on subjective rating scales and two-dimensional photography, which lack depth information. This study aimed to assess the clinical utility of a laser-based three-dimensional (3D) imaging approach for objective quantitative evaluation of glabellar lines in adults undergoing botulinum toxin treatment. **Methods:** A laser-based 3D imaging system was used to quantitatively measure glabellar line morphology. System accuracy for area, perimeter, volume, and depth was evaluated using standardized physical models. In a prospective observational study, 31 adults with moderate-to-severe glabellar lines undergoing routine botulinum toxin treatment were assessed at baseline, day 7, and week 4. Quantitative 3D measurements were compared with clinician- and participant-reported severity scores, as well as patient satisfaction and Global Rating of Outcome (GRO) scores. **Results:** The 3D imaging measurements demonstrated high geometric measurement precision, with errors ≤2% for area, perimeter, and volume, and ≤0.5 mm for depth. Significant reductions in wrinkle depth were observed after treatment. Quantitative 3D measurements showed moderate correlations with clinician-reported scores (r = 0.53–0.54) and participant-reported scores (r = 0.59–0.66). Improvement rates derived from 3D measurements were positively correlated with patient satisfaction and GRO scores. **Conclusions:** Laser-based 3D imaging provides an objective and quantitative approach for evaluating glabellar lines and treatment response to botulinum toxin. This method may complement conventional clinical assessments and support further validation in larger clinical studies.

## 1. Introduction

Accurate and reproducible objective evaluation of skin surface morphology is essential in aesthetic medicine [1,2]. With the growing demand for cosmetic procedures such as neuromodulator-based wrinkle reduction, soft-tissue filler injections, fat-dissolving treatments, and facial contouring, there is an increasing need for reliable and quantitative methods to assess treatment outcomes. However, most existing evaluations continue to rely on subjective clinical rating scales, including the Investigator Global Assessment for Frown Wrinkle Severity (IGA-FWS) [3], Rao–Goldman’s 5-point Wrinkle Scale [4], and Glabellar Line Severity Scale (GLSS) [5], all of which are affected by inter- and intra-rater variability. These limitations highlight the need for objective and quantitative imaging methods in dermatology.

Although two-dimensional photography remains widely adopted owing to its ease of use and accessibility, it lacks depth information and is highly susceptible to environmental and operator variability [6]. Efforts to improve photographic precision through computational enhancements or more advanced imaging setups have encountered obstacles such as technical complexity, limited reproducibility, and poor integration into clinical workflows [7,8,9]. Dermoscopy, although valuable for enhancing surface visualization in clinical practice, offers only a limited field of view and lacks the depth information required for quantitative assessment [10].

Three-dimensional (3D) imaging technologies have emerged as promising tools for objective dermatologic evaluation, providing measurable surface data and improved diagnostic support. Systems based on stereophotogrammetry, such as 3D LifeViz^®^ and Vectra^®^, have demonstrated utility in tracking structural changes after botulinum toxin or filler injections [11,12]. However, these platforms construct 3D models from 2D images and do not directly capture raw point cloud data. Structured light systems (e.g., Antera 3D^®^ and PRIMOSCR) provide higher surface resolution but are often limited by prolonged acquisition times and high sensitivity to ambient lighting [13,14,15,16]. Furthermore, both stereophotogrammetry and structured light technologies are limited by proprietary hardware and software, limiting system flexibility and obstructing data sharing.

Laser-based 3D imaging, which utilizes triangulation to generate raw point cloud data, has several advantages over existing methods, including faster acquisition times, greater robustness under inconsistent lighting, and greater measurement accuracy [17]. Despite these benefits, their clinical application in dermatology remains underexplored. Proprietary hardware–software ecosystems further limit the integration of laser-based 3D data into precision medicine tools and AI-assisted diagnostic frameworks [18]. To address these limitations, this study proposes an open and modular 3D evaluation system built around a conventional laser-based 3D camera, combined with open-source analytical software. Unlike proprietary imaging platforms, the proposed system enables direct access to raw point cloud data, customizable preprocessing workflows, and flexible depth extraction pipelines. The novelty of this approach lies not only in hardware configuration but also in its transparent software architecture and clinically deployable analytical workflow. The system was subjected to calibration and clinical validation to assess its feasibility in addressing current imaging limitations, particularly in capturing treatment-related morphological changes, such as glabellar line depth variations.

## 2. Materials and Methods

### 2.1. 3D Skin Evaluation System Design and Components

**Figure 1:**
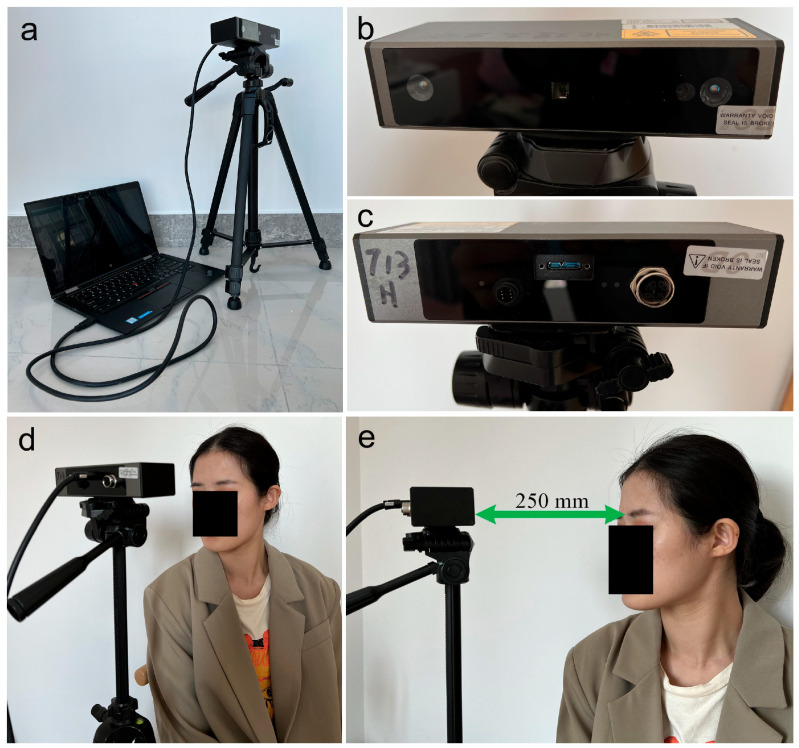
Hardware configuration and clinical application of the laser-based 3D imaging system. (**a**) The 3D camera mounted on a tripod with the data cable connected. (**b**) Frontal view of the 3D camera. (**c**) Rear view of the 3D camera. (**d**) A subject undergoing 3D image acquisition with the head slightly rotated toward the camera. (**e**) The camera was positioned directly in front of the glabellar region at a fixed horizontal distance of 250 mm.

A compact laser-based 3D imaging system was developed to quantitatively evaluate skin surface morphology. The setup consisted of a commercially manufactured laser-based 3D camera (Surface 120, Revopoint 3D Technologies Co., Ltd., Xi’an, China) connected via USB to a standard laptop. The device was commercially available at the time of study and was used in its standard factory configuration without hardware modification. The camera was positioned approximately 250 mm from the skin, and its optical axis was set perpendicular to the region of interest to minimize geometric distortion (Figure 1). A Class I eye-safe laser pattern was projected onto the skin to generate depth data, which was captured as 3D point clouds in Polygon File Format (PLY) using proprietary 3DViewer software (v3.2.0). Open-source tools were employed for post-capture processing and analysis. MeshLab (v2022.02) was used for surface reconstruction and smoothing and to calculate the area and perimeter. CloudCompare (v2.12) was used for volume calculation through its “Compute 2.5D Volume” function. The height and depth of the surface features (e.g., wrinkles or lesions) were evaluated by establishing a reference plane with CloudCompare’s MPlane plugin, allowing for objective and reproducible metric assessments. Each 3D frame was captured within 2–3 s to reduce motion artifacts and improve the measurement reliability. This compact system was employed in both the calibration and clinical experiments.

### 2.2. Calibration Experiments for Measurement Accuracy and Precision

To evaluate the measurement accuracy and precision of the 3D imaging system, a series of calibration experiments was conducted using physical models with predefined geometries. For planar calibration (area and perimeter), 20 square patches ranging in size from 5 to 100 mm per side were scanned and analyzed using MeshLab. Volumetric accuracy was assessed using bell-shaped Blu Tack^®^ models (100–2000 mm^3^), with actual volumes derived from the measured mass and known material density. These were compared with the volumes calculated from the 3D point clouds in CloudCompare. To examine the accuracy of the depth measurement, stacks of microscope cover glasses with known thicknesses (0.17–6.12 mm) were imaged, and the vertical step heights were measured relative to a reference plane. These experiments quantitatively and qualitatively confirmed the geometric measurement performance of the system under controlled conditions prior to its use in clinical morphological assessment.

### 2.3. Measurement and Depth Analysis of Glabellar Lines

**Figure 2:**
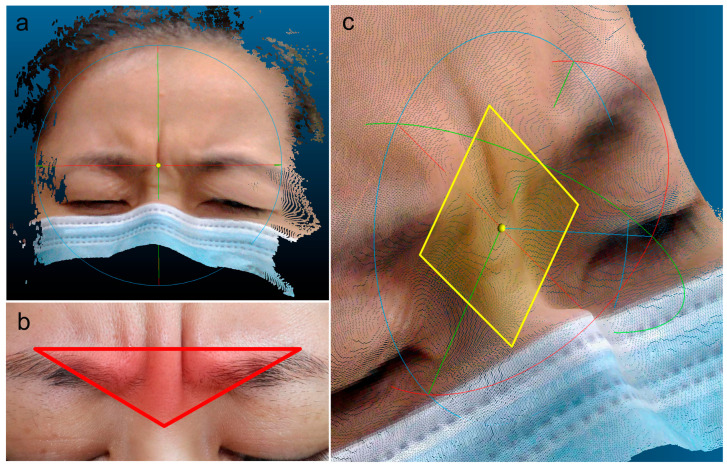
Preprocessing and leveling of the glabellar region in CloudCompare. (**a**) The raw point cloud was imported into CloudCompare, and non-essential regions were removed using the segmentation tool to isolate the glabella. (**b**) Three points were selected within the triangular area defined by the nasion and the two eyebrow peaks to generate a local reference plane (red region). (**c**) The glabellar plane was successfully re-leveled and aligned parallel to the internal XY plane of CloudCompare (yellow surface).

Depth analysis of glabellar lines was performed on a representative clinical case using CloudCompare and its MPlane plugin. A schematic overview of the analytical workflow is provided in Figure S1. The raw 3D point cloud was first imported, and regions not relevant to the analysis, such as hair and areas beneath the eyes, were removed using the segmentation tool to isolate the glabellar area (Figure 2a). The point cloud was then reoriented by selecting three anatomical landmarks: the nasion and peaks of both eyebrows (Figure 2b). These points defined a local reference plane aligned parallel to CloudCompare’s internal XY plane, allowing for a consistent depth assessment (Figure 2c).

**Figure 3:**
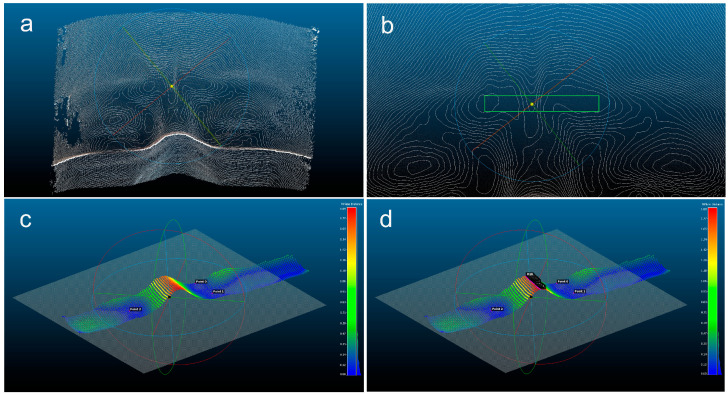
Glabellar line selection and depth extraction in CloudCompare. (**a**) Facial contour plot generated from the re-leveled point cloud. (**b**) Rectangular region selected along the uppermost contour (green) to include the deepest glabellar furrows. (**c**) Zero plane created by selecting three low points; elevation visualized using a color ramp. (**d**) Line of points from the red zone extracted for glabellar line depth analysis.

A facial contour plot was generated from the re-leveled point cloud (Figure 3a). A rectangular region over the glabella was selected along the uppermost contour to include the deepest grooves (Figure 3b). Using the MPlane plugin, three lower points within the selected region were used to establish a zero-reference plane. This produced a color-coded elevation map, where red represented the deepest areas. A line of points along the most sunken region was extracted for the measurement (Figure 3c,d). For each subject at each time point, all depth values from the extracted point cloud were analyzed, with the median value reported as the final quantitative measurement of the glabellar wrinkle depth.

Point selection and region-of-interest placement were performed manually by a trained operator following standardized anatomical guidelines. The rectangular glabellar region was consistently positioned relative to anatomical landmarks to ensure comparable coverage across subjects and time points. To minimize point cloud noise, standard filtering and smoothing functions within CloudCompare were applied prior to depth extraction.

### 2.4. Clinical Evaluation Design

An observational clinical evaluation was conducted in adults with glabellar lines at Beijing Tsinghua Changgung Hospital. Thirty-one adults (27 females and four males; age range, 27–65 years) undergoing cosmetic botulinum toxin treatment were included. The botulinum toxin treatment and associated clinical evaluation were conducted in accordance with the Declaration of Helsinki and were approved by the Ethics Committee of Beijing Tsinghua Changgung Hospital. Written informed consent was obtained from all participants. All participants were of Han Chinese ethnicity and exhibited moderate-to-severe glabellar lines at baseline. Eligible individuals had no recent history of neuromuscular disorders and had not received botulinum toxin treatment within six months prior to enrollment. Participants received a total of 20 units of botulinum toxin type A, injected at five standardized sites in the glabellar region. One injection (4 units) was administered centrally into the procerus muscle, and two injections (4 units each) were placed on each side to target the medial and lateral aspects of the corrugator supercilii muscle.

Clinical assessments and 3D imaging were conducted at baseline (Day 0), Day 7 (±1 day), and Week 4 (±1 week) post-injection. The severity of glabellar lines during maximal frowning was independently evaluated by a blinded dermatologist and by the participants themselves using a validated 4-point ordinal scale (0–3), where higher scores indicated greater wrinkle severity. A score of 0 represented no visible glabellar lines; 1 indicated visible but mild lines; 2 corresponded to clearly visible lines with the groove bottom still discernible; and 3 denoted severe lines with deep grooves where the bottom was no longer visible. For inclusion, moderate-to-severe glabellar lines were defined as a baseline score of 2 or 3.

3D images of the glabellar region at maximal frown were captured from a distance of approximately 250 mm under standardized posture conditions. Image data were analyzed in CloudCompare and MeshLab by a blinded analyst to quantify maximum wrinkle depth. These depth values were subsequently compared with clinician-assessed and participant-reported wrinkle severity scores to evaluate correlation.

In addition to severity ratings, all participants completed two self-assessment questionnaires at Day 7 and Week 4: a 9-point Global Rating of Outcome (GRO) scale (Table S1) and a 7-point Participant Satisfaction (PS) scale (Table S2). Objective wrinkle improvement was calculated for each participant based on 3D-derived depth measurements using the following formula:

$$3D-Measured\;Wrinkle\;Improvement\;Rate\;(\%)=\frac{{Depth}_{D0}-{Depth}_{D7\;or\;W4}}{{Depth}_{D0}}\times 100$$

The 3D-measured wrinkle improvement rates were then compared with GRO and PS scores to assess consistency across objective and subjective measures.

### 2.5. Statistical Analysis

Data normality was assessed using both the D’Agostino–Pearson omnibus test and the Shapiro–Wilk test. Pearson correlation analysis was used to compare area, perimeter, and volume measurements with standardized reference values, whereas Spearman correlation analysis was used for depth measurements due to their non-normal distribution. Spearman correlation analysis was also applied to evaluate the associations between 3D-measured wrinkle depth and clinical severity scores, as well as between 3D-measured wrinkle improvement rates and GRO/PS scores. For both types of correlations, the coefficients (r) ranged from 0 to 1, with higher values reflecting stronger agreement. Given the limited sample size, Fisher’s exact test was used to analyze differences in clinical assessment outcomes. Comparisons of 3D data across Day 0, Day 7, and Week 4 post-treatment were performed using the Kruskal–Wallis test, followed by Dunn’s multiple comparisons test. Linear regression analysis was conducted to assess the relationship between 3D measurements and reference standards, as well as between 3D wrinkle depth and clinical severity scores. All statistical analyses were performed using GraphPad Prism (version 10.4.0). Linear regression plots were generated in Python (version 3.11.5) using Visual Studio Code (version 1.101.2).

## 3. Results

### 3.1. System Calibration Results

**Figure 4:**
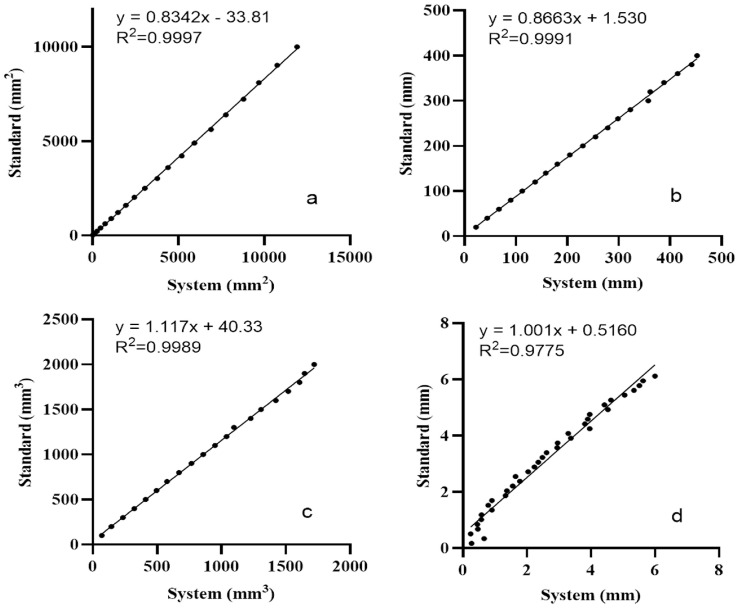
Linear regression plots for calibration results. (**a**) Area, (**b**) perimeter, (**c**) volume, and (**d**) depth measurements calculated by the 3D evaluation system show excellent linear agreement with corresponding standardized values.

The laser-based 3D imaging system exhibited excellent geometric measurement accuracy and reproducibility in measuring the surface area, perimeter, volume, and depth, with correlation coefficients of r = 0.9998, 0.9995, 0.9995, and 0.9928, respectively (*p* < 0.0001). Corresponding regression plots are presented in Figure 4. For the area and perimeter, deviations remained minimal when evaluating objects smaller than 625 mm^2^ and 100 mm, respectively. Volume measurements showed a slight underestimation at the higher end, whereas depth values displayed increased variability below 1 mm. Across all calibration trials, measurement errors were consistently within 1–2% for area, perimeter, and volume and within ±0.5 mm for depth. These results confirm the reliability of the system for detecting fine topographic variations in dermatological assessments, including wrinkle analysis and lesion profiling, supporting its application in the clinical evaluation of glabellar lines.

### 3.2. Improvement in Glabellar Lines After Treatment

**Figure 5:**
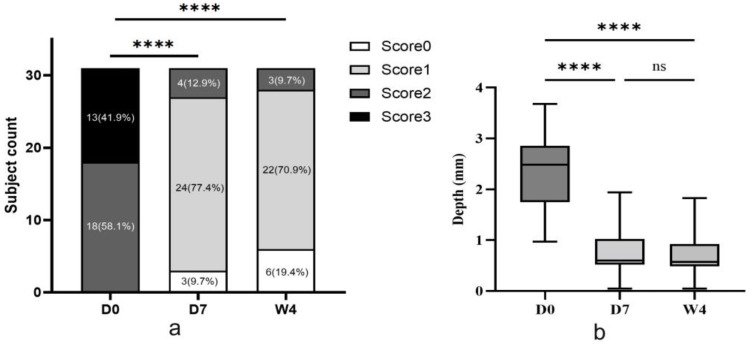
(**a**) Distribution of patients by glabellar line severity scores (0–3) at each time point. Data are presented as number of subjects (percentage). (**b**) Mean glabellar wrinkle depth measured by 3D imaging across time points. D0 = pre-treatment; D7 = 7 days post-treatment; W4 = 4 weeks post-treatment. **** *p* < 0.0001; ns = not significant.

All 31 patients completed the 4-week follow-up period without any serious adverse events. At baseline, most individuals exhibited moderate-to-severe glabellar lines with frown severity scores of 2 or 3. By Day 7, improvement was recorded in 27 of the 31 patients, and by Week 4, 28 of the 31 patients had improved to a score of 0 or 1. Notably, 6 individuals showed no visible glabellar lines (score 0) at Week 4. The mean frown severity scores were significantly reduced on both Day 7 and Week 4 (*p* < 0.0001) (Figure 5). These results are consistent with the known pharmacodynamic profile of botulinum toxin. Detailed scores for each time point are presented in Table S3.

### 3.3. 3D Wrinkle Depth Measurements

Quantitative analysis of the glabellar wrinkle depth during frowning was performed at baseline, Day 7, and Week 4 using 3D imaging. As shown in Table S3, all 31 patients exhibited a reduction in wrinkle depth by Day 7 and Week 4, although the degree of improvement varied among the individuals. This variation highlights the inter-individual differences in treatment responses. A comprehensive summary of the depth changes is presented in the box-and-whisker plots (Figure 5). Statistical analysis confirmed that wrinkle depth was significantly lower at both Day 7 and Week 4 than at baseline (*p* ≤ 0.0001), while no significant difference was observed between Day 7 and Week 4.

### 3.4. Comparison of 3D Measurements with Subjective Assessments

**Figure 6:**
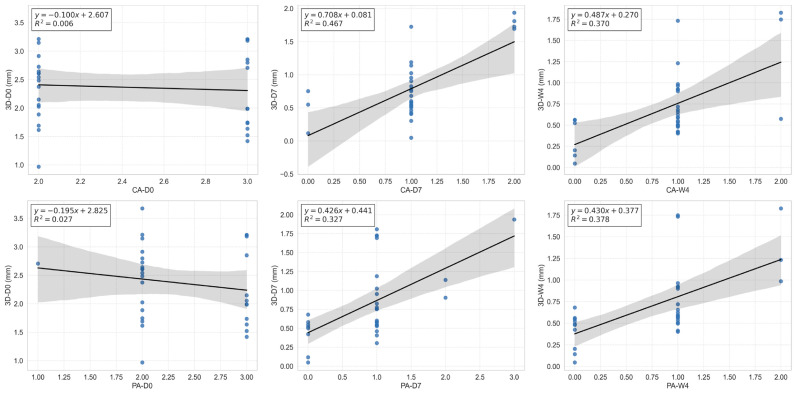
Correlations between 3D wrinkle depth and clinical or participant self-assessed severity scores. CA-D0/D7/W4: Clinical assessments at pre-treatment (D0), 7 days (D7), and 4 weeks (W4) post-treatment. PA-D0/D7/W4: Participant assessments at pre-treatment (D0), 7 days (D7), and 4 weeks (W4) post-treatment. 3D-D0/D7/W4: 3D evaluations at pre-treatment (D0), 7 days (D7), and 4 weeks (W4) post-treatment. Solid lines represent linear regression fits, and shaded areas indicate 95% confidence intervals of the regression lines. R^2^ values are shown in each panel.

To evaluate the consistency between the objective 3D imaging data and subjective assessments, all 3D frown-related variables were plotted against the corresponding clinical or participant self-assessed severity scores (Figure 6). At Day 0, no significant correlations were observed between 3D wrinkle depth and either clinical or participant severity scores. At Day 7, 3D-measured glabellar depth showed moderate positive correlations with both clinician-rated severity (r = 0.5327, *p* = 0.002) and participant-reported severity (r = 0.5855, *p* = 0.0005). These associations persisted at Week 4, with similar correlation strength (r = 0.5376, *p* = 0.0018; r = 0.6592, *p* < 0.0001, respectively). Moreover, the 3D-measured wrinkle improvement rate was positively correlated with PS scores at both time points (Day 7: r = 0.4070, *p* = 0.0231; Week 4: r = 0.4020, *p* = 0.0250), as well as with participant-reported GRO scores (Day 7: r = 0.4850, *p* = 0.0057; Week 4: r = 0.4892, *p* = 0.0052). These findings collectively support the validity of 3D imaging as an objective tool for assessing both clinically observable and participant-perceived treatment efficacy.

## 4. Discussion

This study presents a preliminary evaluation of an open-architecture laser-based 3D imaging system for quantitative assessment of glabellar lines. By capturing raw 3D point cloud data and processing them with open-source software, the system enabled objective wrinkle depth measurements before and after botulinum toxin treatment. The modular design using commercially available components may improve practical accessibility relative to proprietary systems.

Calibration demonstrated high measurement accuracy, with depth and geometric errors falling within acceptable ranges for facial aesthetic assessment [19,20,21]. Nevertheless, given a measurement accuracy of approximately ±0.5 mm, the system is more appropriately suited for longitudinal monitoring of relative changes within the same individual rather than precise absolute quantification of very fine micro-wrinkle depths. In the clinical study, reductions in wrinkle depth observed on 3D imaging were consistent with expected pharmacodynamic effects of botulinum toxin. Moderate correlations with clinician- and participant-reported severity scores at post-treatment time points suggest that 3D depth metrics capture clinically recognizable changes. Collectively, these findings support the positioning of 3D imaging primarily as a research and complementary assessment tool that supports, rather than replaces, established clinical rating scales.

**Figure 7:**
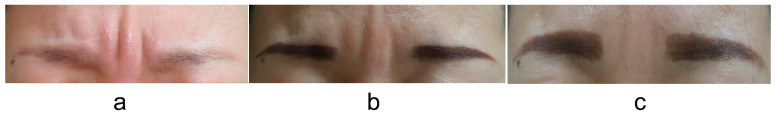
Representative example demonstrating discordance between clinical severity grading and quantitative 3D depth measurement. Standardized 2D photographs of a subject in the frown position at (**a**) pre-treatment, (**b**) 7 days post-treatment, and (**c**) 4 weeks post-treatment. The clinical investigator assigned an unchanged severity score of 2 at all time points. In contrast, 3D analysis revealed a progressive reduction in glabellar line depth (median values: 2.642 mm, 1.693 mm, and 0.607 mm, respectively).

Notably, Figure 7 illustrates a representative case in which measurable improvement in 3D-derived wrinkle depth was observed despite minimal change in clinical severity grading. This discrepancy highlights the increased sensitivity of quantitative 3D imaging in detecting treatment-related morphological changes that may not be fully captured by ordinal visual assessment scales. Such findings reinforce the value of objective depth profiling as a complementary tool alongside conventional clinical evaluation methods.

At baseline, however, 3D wrinkle depths did not correlate significantly with clinical severity scores. This finding is most likely attributable to range restriction in this cohort, as all participants were intentionally recruited with only moderate-to-severe glabellar lines (clinical scores 2–3). Because the cohort spanned only a narrow severity range, both clinical scores and depth measurements showed limited variability, which likely attenuated correlation estimates. This restricted-score effect likely accounts for the lack of significant baseline correlation between quantitative 3D depth metrics and clinical severity scores. At post-treatment follow-up time points, relatively stronger correlations were observed between quantitative 3D depth metrics and subjective severity scores. However, the overall moderate correlations may reflect the inherent limitations of ordinal visual grading scales, which lack the resolution to capture subtle morphological changes detectable by objective 3D measurements.

Beyond the evaluation of botulinum toxin outcomes, this system has potential applicability across aesthetic procedures that require precise assessment of surface and depth features. Examples include soft-tissue augmentation and facial contour analysis. In future aesthetic studies, 3D-derived metrics may serve as objective endpoints, such as localized volume change or depth reduction, which may help improve the consistency and reproducibility of outcome evaluation. For patients, 3D visualization may also support communication by providing clear and tangible representations of treatment-related changes.

Compared with existing commercial technologies, the system demonstrates potential advantages in portability and platform flexibility. Commercial systems often rely on fixed imaging setups, proprietary software, and tightly controlled lighting conditions [22,23]. The present system uses open-source tools and operates under ambient lighting conditions, allowing flexible and transparent data processing. A theoretical comparison of key system characteristics is provided in Table S4 to contextualize its positioning relative to established commercial technologies. Direct empirical benchmarking against established commercial systems was beyond the scope of the present study and remains an area for future investigation.

This study has several limitations. The clinical cohort was small, derived from a single center, and ethnically homogeneous, consisting entirely of Han Chinese participants and predominantly females. This limited demographic diversity may restrict the generalizability of the findings, particularly given potential variations in skin properties and wrinkle characteristics across different ethnicities and skin phototypes. All measurements in this study were performed by a single trained analyst using standardized segmentation and alignment protocols to ensure internal consistency. However, formal intra- or inter-operator variability assessment was not conducted in this preliminary study and is acknowledged as a limitation. Additionally, at the hardware level, the current camera resolution may not reliably capture micro-wrinkles smaller than 0.2 mm.

Future work will focus on improving sensor resolution, automating segmentation and alignment, including participants across the full range of wrinkle severity, and evaluating performance against commercial systems [24]. Larger, multicenter studies are needed to assess reproducibility, inter-rater reliability, and the potential role of 3D-derived measurements as objective endpoints in aesthetic clinical research. In parallel, further development of the analytical pipeline may incorporate artificial intelligence-based frameworks to facilitate automated wrinkle detection, segmentation, and longitudinal outcome prediction.

Overall, this study provides an initial demonstration of the feasibility of laser-based 3D wrinkle quantification. The findings are encouraging, but further refinement and broader validation are necessary before this system can be confidently applied in clinical research or routine aesthetic practice.

## 5. Conclusions

Laser-based 3D imaging provides an objective and quantitative approach for evaluating glabellar lines. The observed correlations with clinical ratings and patient-reported outcomes suggest that it may serve as a complementary tool to support standard clinical assessment in aesthetic practice. Further validation in larger and more diverse cohorts is warranted.

## Data Availability

The data presented in this study are available on request from the corresponding author due to privacy of the subjects involved.

## Supplementary Materials

The following supporting information can be downloaded at https://www.mdpi.com/article/10.3390/diagnostics16050679/s1, Figure S1: Workflow diagram illustrating the 3D point cloud processing and depth extraction pipeline performed using CloudCompare; Table S1: Self-Reported Global Rating of Outcome Scale (9 Points); Table S2: Participant Self-Reported Satisfaction Scale (7 Points); Table S3: Glabellar line severity scores and 3D-measured wrinkle depth at Day 0, Day 7, and Week 4, with individual percentage depth changes; Table S4: Theoretical comparison of the proposed laser triangulation imaging system with stereophotogrammetry and structured light platforms.

## Abbreviations

The following abbreviations are used in this manuscript:

3D	Three-dimensional
GRO	Global rating of outcome
IGA-FWS	Investigator global assessment for frown wrinkle severity
GLSS	Glabellar line severity scale
PLY	Polygon file format
PS	Participant satisfaction
CA	Clinical assessment
PA	Participant assessment
