# Supplementary material for: Utility of 3D Imaging in the Objective Evaluation of Glabellar Lines Following Botulinum Toxin Treatment
Source: Diagnostics (Basel). 2026 Feb 26;16(5):679. doi: 10.3390/diagnostics16050679 (PMC12984208; doi:10.3390/diagnostics16050679)
Supplement: Supplementary file 1 [file diagnostics-16-00679-s001.zip › Supplementary Figure S1.pdf]

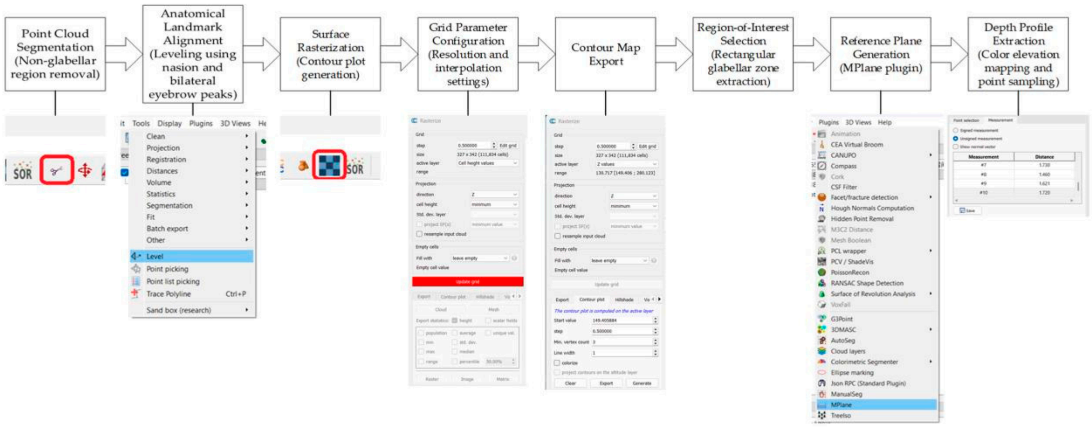

Supplementary Figure S1. Workflow diagram illustrating the 3D point cloud processing and depth extraction pipeline performed using CloudCompare (version 2.12). Sequential steps include segmentation of non-relevant facial regions, anatomical landmark alignment, surface rasterization and contour generation, grid parameter configuration, contour export, region-of-interest selection, reference plane generation using the MPlane plugin, and quantitative depth profile extraction via elevation mapping and point sampling.
