# Supplementary material for: Utility of 3D Imaging in the Objective Evaluation of Glabellar Lines Following Botulinum Toxin Treatment
Source: Diagnostics (Basel). 2026 Feb 26;16(5):679. doi: 10.3390/diagnostics16050679 (PMC12984208; doi:10.3390/diagnostics16050679)
Supplement: Supplementary file 1 [file diagnostics-16-00679-s001.zip › Supplementary Table S1.pdf]

Supplementary Table S1. Self-Reported Global Rating of Outcome Scale (9 Points)

Standard	Score	Improvement	Description
Improvement Evaluation	+4	Total Improvement	~ 100 % Improvement
	+3	Significant Improvement	~ 75 % Improvement
	+2	Moderate Improvement	~ 50 % Improvement
	+1	Minor Improvement	~ 25 % Improvement
	0	No Change	No change
	-1	Minor Deterioration	~ 25 % Deterioration
	-2	Moderate Deterioration	~ 50 % Deterioration
	-3	Significant Deterioration	~ 75 % Deterioration
	-4	Total Deterioration	~ 100 % Deterioration
