# Supplementary material for: Utility of 3D Imaging in the Objective Evaluation of Glabellar Lines Following Botulinum Toxin Treatment
Source: Diagnostics (Basel). 2026 Feb 26;16(5):679. doi: 10.3390/diagnostics16050679 (PMC12984208; doi:10.3390/diagnostics16050679)
Supplement: Supplementary file 1 [file diagnostics-16-00679-s001.zip › Supplementary Table S2.pdf]

Supplementary Table S2. Participant Self-Reported Satisfaction Scale (7 Points)

Standard	Score	Satisfaction
Patient Satisfaction Evaluation	7	Very Satisfied
	6	Satisfied
	5	Slightly Satisfied
	4	Neither Satisfied nor Dissatisfied
	3	Slightly Dissatisfied
	2	Dissatisfied
	1	Very Dissatisfied
