# Supplementary material for: Utility of 3D Imaging in the Objective Evaluation of Glabellar Lines Following Botulinum Toxin Treatment
Source: Diagnostics (Basel). 2026 Feb 26;16(5):679. doi: 10.3390/diagnostics16050679 (PMC12984208; doi:10.3390/diagnostics16050679)
Supplement: Supplementary file 1 [file diagnostics-16-00679-s001.zip › Supplementary Table S3.pdf]

Supplementary Table S3. Glabellar line severity scores and 3D-measured wrinkle depth at Day 0, Day 7, and Week 4, with individual percentage depth changes.

	Day 0		Day 7			Week 4		
	Severity	Wrinkle Depth	Severity	Wrinkle Depth	Improvement	Severity	Wrinkle Depth	Improvement
ID	Score	(mm)	Score	(mm)	Rate (%)	Score	(mm)	Rate (%)
2	2	3.758	0	0.548	85.4	0	0.525	86.0
3	2	0.969	1	0.430	55.6	1	0.408	57.9
4	2	2.559	1	0.049	98.1	0	0.046	98.2
6	3	3.246	1	0.529	83.7	1	0.593	81.7
7	2	2.727	1	0.954	65.0	1	0.921	66.2
8	3	1.637	1	0.502	69.3	1	0.495	69.8
10	3	2.707	1	1.025	62.1	1	0.688	74.6
11	2	2.914	1	0.603	79.3	0	0.563	80.7
12	3	1.745	1	0.543	68.9	1	0.656	62.4
13	2	2.057	1	0.582	71.7	1	0.533	74.1
14	3	2.798	0	0.752	73.1	1	0.666	76.2
15	2	2.375	1	0.583	75.5	1	0.681	71.3
16	3	1.523	1	0.460	69.8	1	0.534	64.9
17	2	1.696	0	0.117	93.1	0	0.143	91.6
18	3	1.986	1	0.681	65.7	0	0.561	71.8
21	3	1.420	1	0.753	47.0	1	0.589	58.5
22	2	1.645	1	0.584	64.5	1	0.485	70.5
23	2	2.542	1	0.916	64.0	1	0.976	61.6
24	2	3.148	2	1.809	42.5	1	0.925	70.6
25	3	1.736	1	1.139	34.4	1	1.232	29.0
26	3	1.991	1	1.189	40.3	1	0.964	51.6
27	3	3.209	2	1.725	46.2	2	1.748	45.5
28	2	2.608	1	0.423	83.8	0	0.203	92.2
29	2	2.614	1	0.826	68.4	1	0.425	83.7
30	2	1.888	1	0.304	83.9	1	0.429	77.3
31	2	2.133	1	0.548	74.3	1	0.525	75.4
32	2	2.055	1	1.755	14.6	1	1.733	15.7
34	2	3.213	1	0.515	84.0	1	0.489	84.8
35	3	2.866	1	0.775	73.0	1	0.900	68.6
37	2	2.642	2	1.693	35.9	2	0.607	77.0
38	3	3.184	2	1.937	39.2	2	1.828	42.6

Note: Severity scores were evaluated by a blinded dermatologist using a validated 4-point ordinal scale (0–3), where higher scores indicated greater wrinkle severity. Depth values are reported in millimeters (mm). Improvement rate (%) for each follow-up visit was calculated as: $(Depth_{baseline} - Depth_{follow\text{-}up}) / Depth_{baseline} \times 100$. All percentages are rounded to one decimal place.
