# Supplementary material for: Utility of 3D Imaging in the Objective Evaluation of Glabellar Lines Following Botulinum Toxin Treatment
Source: Diagnostics (Basel). 2026 Feb 26;16(5):679. doi: 10.3390/diagnostics16050679 (PMC12984208; doi:10.3390/diagnostics16050679)
Supplement: Supplementary file 1 [file diagnostics-16-00679-s001.zip › Supplementary Table S4.pdf]

Supplementary Table S4. Theoretical comparison of the proposed laser triangulation imaging system with stereophotogrammetry and structured light platforms.

	**Laser-based 3D imaging (Proposed System)**	**Stereophotogrammetry**	**Structured Light**
Imaging principle	Laser triangulation point-cloud acquisition	Multi-camera photogrammetric reconstruction	Projected light pattern deformation
Spatial resolution	High (sub-millimeter depth profiling)	Moderate	Moderate–high
Depth accuracy	±0.5 mm (calibration validated)	Surface approximation dependent	±0.3–0.8 mm (system dependent)
Acquisition time	Rapid	Rapid	Rapid
Portability	High (modular configuration)	Low (fixed multi-camera rigs)	Moderate
Hardware cost	Accessibility-oriented / modular	High	Moderate–high
Software ecosystem	Open-source compatible	Proprietary	Proprietary
Raw data accessibility	Full point-cloud access	Limited	Limited
Workflow flexibility	High (customizable processing)	Low	Moderate
Lighting requirements	Ambient lighting compatible	Controlled lighting preferred	Structured projection required
