# Supplementary figures and images for: Utility of 3D Imaging in the Objective Evaluation of Glabellar Lines Following Botulinum Toxin Treatment
Source: Diagnostics (Basel). 2026 Feb 26;16(5):679. doi: 10.3390/diagnostics16050679 (PMC12984208; doi:10.3390/diagnostics16050679)

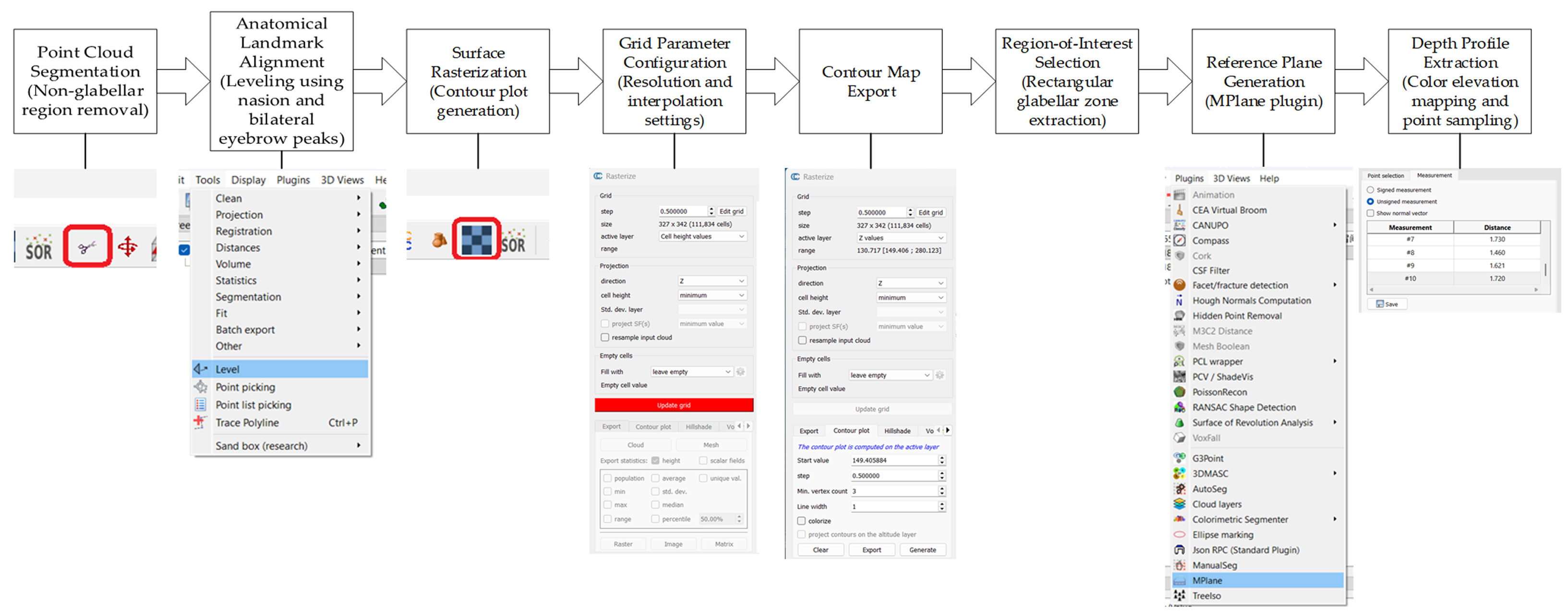

Supplement: Supplementary file 1 [file diagnostics-16-00679-s001.zip › Supplementary Figure S1.jpg]
